# Effects of Caloric Restriction and Rope-Skipping Exercise on Cardiometabolic Health: A Pilot Randomized Controlled Trial in Young Adults

**DOI:** 10.3390/nu13093222

**Published:** 2021-09-16

**Authors:** Zhaoxie Tang, Yingan Ming, Miao Wu, Jiajia Jing, Suhua Xu, Hailin Li, Yanna Zhu

**Affiliations:** 1 Department of Maternal and Child Health, School of Public Health, Sun Yat-sen University, Guangzhou 510080, China; tangzhx5@mail2.sysu.edu.cn (Z.T.); wumiao6@mail2.sysu.edu.cn (M.W.); jingjj3@mail2.sysu.edu.cn (J.J.); xush59@mail2.sysu.edu.cn (S.X.); lihlin29@mail2.sysu.edu.cn (H.L.); 2 Department of Physical Education, Sun Yat-sen University, Guangzhou 510275, China; mingya@mail.sysu.edu.cn; 3 Sun Yat-sen Global Health Institute, Institute of State Governance, Sun Yat-sen University, Guangzhou 510080, China

**Keywords:** caloric restriction, rope-skipping, exercise, cardiometabolic health, obesity

## Abstract

The aim of this study is to investigate the effects of calorie restriction (CR), rope-skipping (RS) exercise, and their joint effects on cardiometabolic health in young adults. An 8-week randomized trial was conducted on 46 undergraduates aged 19–21 y from South China. The participants were randomized into the following three groups: Calorie restriction (CR) group (*n* = 14), Rope-skipping (RS) group (*n* = 14), and CR plus RS (CR–RS) group (*n* = 12). At both allocation and the end of the intervention, data on anthropometry, serum metabolic, and inflammatory markers were collected. A total of 40 participants completed the intervention and were included in the analysis. After the 8-week intervention, the participants from the CR group and the CR–RS group reduced in body weight (−1.1 ± 1.7 kg, −1.3 ± 2.0 kg), body mass index (−0.4 ± 0.6 kg/m^2^, −0.5 ± 0.7 kg/m^2^), body fat percentage (−1.2 ± 1.6%, −1.7 ± 1.8%), and body fat mass (−1.1 kg (−2.2, −0.3), −1.1 kg (−2.5, −0.4)) compared to the baseline (*p* < 0.05 or *p* = 0.051). For metabolic and inflammatory factors, the participants in the CR–RS group showed significant decreases in low density lipoprotein cholesterol (−0.40 mmol/L) and interleukin-8 (−0.73 mmol/L). While all the above markers showed no significant difference among the groups after intervention, in the subgroup of overweight/obese participants (*n* = 23), the CR–RS group had significantly lower blood pressure, fasting insulin, homeostatic model assessment of insulin resistance, tumor necrosis factor-α, and interleukin-8 levels than the CR or RS groups (*p* < 0.05). In conclusion, both CR and CR–RS could reduce weight and improve body composition in young adults. More importantly, in those with overweight or obesity, CR–RS intervention might be superior to either CR or RS in improving cardiometabolic health.

## 1. Introduction

Cardiovascular (CV) disease is the leading cause of morbidity, disability, and death worldwide, and CV risk factors generally include a series of factors associated with cardiometabolic diseases [1,2]. Previous studies showed that certain CV risk factors were closely associated with an increased risk of developing type 2 diabetes and other CV diseases [3], and prospective studies further confirmed that CV risk factors during early adulthood contributed to the CV morbidity and mortality in later life.

Calorie restriction (CR) without malnutrition has been proven effective in facilitating weight loss and improving cardiometabolic health in a variety of animal species [4,5,6]. A study conducted on rhesus monkeys reported that early onset CR reduced the monkeys’ risk of developing and dying of CV diseases by even more than 50% [7,8]. However, in humans, the effect of CR on CV risk factors has been controversial and is not well understood, especially in those generally healthy young adults. The CALERIE (Comprehensive Assessment of Long-term Effects of Reducing Intake of Energy) phase one trial was the first controlled clinical trial of CR in non-obese healthy humans [9]. Additionally, it suggested that the 25% CR diet for 6 months made a 29% reduction in the ten-year risk for CV disease, while various CV risk factors (e.g., blood pressure, low density lipoprotein cholesterol (LDL-C), high density lipoprotein cholesterol (HDL-C), C-reactive protein, and tumor necrosis factor-α (TNF-α)) were not affected by CR [10,11]. In the CALERIE phase two trials, 2-years of CR significantly improved the body composition, cardiometabolic risk markers, and markers of inflammation in young and middle-aged, healthy non-obese participants [12,13,14].

Exercise has long been considered as a good approach to prevent noncommunicable diseases and improve cardiometabolic health [15,16]. For instance, a number of studies between 2010 and 2020 showed that an appropriate amount of exercise improved the cardiometabolic health in individuals with normal-weight, overweight, obesity, metabolic syndrome, or diabetes mellitus [16,17]. In those studies, though the exercise interventions were differently designed, moderate- to high-intensity exercise was mostly used, and generally, the intervention duration varied in different studies. For example, the results of a study by Julia Otten and her colleagues in 2019 showed that moderate-intensity aerobic exercise and high-intensity intermittent training significantly reduced the myocardial triglycerides levels in overweight and obese individuals with type 2 diabetes mellitus [18]. Rope-skipping (RS) exercise was proven to be an affordable, easily accessible, and fun exercise modality that has a high exercise adherence rate in students [19]. Additionally, a recent randomized control trial proved that RS exercise could improve CV risk factors of obese adolescent girls, including body fat percent, waist circumference (WC), blood pressure, blood glucose, insulin levels, and homeostatic model assessment of insulin resistance (HOMA-IR) [20].

Despite the existing evidence on the positive effects of CR and exercise on CV health, prior studies mainly focused only on CR or exercise, few studies have implied both interventions in one study. The joint effects of the two intervention strategies remain unclear. The present study aimed to investigate the effects of CR, RS exercise, and their joint interventions on cardiometabolic health in undergraduate students from South China.

## 2. Materials and Methods

### 2.1. Study Design

This was a single-blind, 8-week randomized trial. Participants were randomized into one of the following 3 groups: CR group, RS group, and CR–RS group.

The experimental protocol was approved by the Ethics Committee of School of Public Health, Sun Yat-sen University, and registered on ClinicalTrials.gov (NCT04275440). All participants gave written informed consent to participate in the trial.

### 2.2. Participants and Randomization

**Figure 1:**
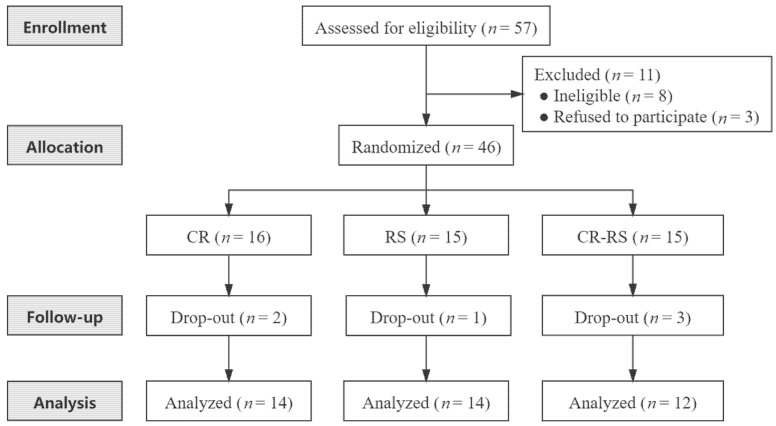
CONSORT flow diagram. CR, calorie restriction; RS, rope-skipping; CR–RS, calorie restriction with rope-skipping.

Participants were recruited from Sun Yat-sen University, Guangzhou, South China. A total of 57 young adults were assessed for eligibility via a questionnaire and body mass index (BMI) assessment (Figure 1), of whom 11 were excluded because they were ineligible (*n* = 8) or refused to participate (*n* = 3). Inclusion and exclusion criteria are described as follows.

Inclusion criteria: second- or third-year undergraduate students in Sun Yat-sen University (age between 19 and 21 years); BMI ≥ 22 kg/m^2^; body weight stable for 3 months prior to the beginning of the study (weight gain or loss <4 kg); and had the time and will to receive the interventions.

Exclusion criteria: currently engaged in other weight-loss studies; with secondary obesity induced by medicine or other diseases; with high blood pressure, diabetes mellitus or other CV diseases; or with contraindication to exercise.

Finally, 46 young adults were included in the study randomized into 3 groups: CR group (*n* = 16), RS group (*n* = 15), and CR–RS group (*n* = 15).

### 2.3. Intervention

Participants in CR group, RS group, and CR–RS group received dietary intervention (calorie restriction), exercise intervention (rope skipping), and diet-exercise joint intervention, respectively. All interventions started at allocation and lasted for 8 weeks. Recruitment started in October 2019 and ended in October 2019. End-line follow-ups started in December 2019 and ended in December 2019.

#### 2.3.1. Dietary Intervention

Dietary intervention in this study was designed as calorie restriction, which means controlling someone’s daily energy intake between 100 and 110% of his or her basal energy expenditure (BEE). At allocation of the study, participants’ BEE was measured using a Body Composition Tester (InBody 230, InBody Co., Ltd., Seoul, Korea), and an energy intake range was calculated for each participant. In the first two weeks of the intervention, participants gradually reduced their daily energy intake to reach the calorie restriction goal and made sure that their daily energy intake remained within the energy intake range in the following 6 weeks of the intervention. During every day of the intervention, participants reported their food intake details on a self-designed application (APP).

In addition, participants who received dietary intervention were also advised to keep a diverse diet, avoid or reduce intake of sugar-sweetened beverages, and allocate the daily energy intake as 30% for breakfast, 40% for lunch, and 30% for dinner.

#### 2.3.2. Exercise Intervention

Exercise intervention was designed as individualized rope skipping exercise, 90 min a time (20 min RS plus 10 min rest, with 3 repetitions), three times a week, arranged by professional physical education (PE) teachers in groups. In the first two weeks, participants were instructed to gradually increase the amount of rope skipping to 1500 each time (3 times/week), and then they maintained this exercise intensity until the end of the intervention. According to the results of physical fitness test at allocation, individualized RS exercise plans were made to make sure that during every time RS exercise was undertaken, the amount of exercise suited a certain participant. Smartwatches (Huawei) as well as smart-skipping ropes were provided for each participant during the study to record their exercise amount.

### 2.4. Data Collection

At both allocation and the end of intervention, data on anthropometry were collected, and blood samples were collected for laboratory tests. During the 8 weeks of intervention, data on calorie restriction and exercise training were recorded by self-designed APP on cellphone, smartwatches, as well as smart-skipping ropes.

#### 2.4.1. Anthropometry

Anthropometric measurements were all performed by trained project members according to standardized methods. Body weight, body fat mass, body fat percentage, and basal metabolic rate were measured using one Body Composition Tester (Inbody 230), with participants wearing light clothing and no shoes. Height was measured using a portable stadiometer (model TZG, China), with the participant in an upright position and shoeless. Height was measured to the nearest 0.1 mm, and body weight was measured to the nearest 0.1 kg. BMI was calculated as one’s weight in kilograms divided by the square of one’s height in meters, and BEE was calculated according to the results of basal metabolic rate from body composition tester. Sitting blood pressure was measured after a five-minute rest, with a validated mercury sphygmomanometer (model XJ1ID, China) and TZ-1 stethoscope, and the cuff size was determined by the mid-upper arm circumference. Blood pressure was recorded as the average of two measurements, with a one-minute interval.

#### 2.4.2. Cardiopulmonary Exercise Testing

The cardiopulmonary exercise testing was conducted at allocation to obtain data on participants’ cardiopulmonary function, in order to make individualized exercise plans in the following intervention. The testing consisted of 800-meter run, 1000-meter run, and RS test. All tests were applied in the standard stadium of Sun Yat-sen University, under the guidance of professional PE teachers, and recorded by stopwatches and smart-skipping ropes. Participants were encouraged to finish the running and RS tests to their best ability, but they were also told that they should stop the tests if feeling dizziness, nausea, chest pain, or other uncomfortable symptom.

#### 2.4.3. Laboratory Tests

At both allocation and the end of intervention, blood samples were collected. After fasting for at least 8 h, 5 mL of venous blood were taken, aliquoted after centrifuged at 3000 rpm (~1000 g) for 10 min and stored at −80 °C before test. The biochemical analysis of blood samples was performed at a biomedical analysis company (KingMed Diagnostics Group Co., Ltd., Guangzhou, China). Relevant laboratory indices are listed as follows.

Metabolic risk markers: Triglyceride (TG), total cholesterol (TC), HDL-C, LDL-C, and uric acid were measured using the enzymatic method. Fasting plasma glucose (FPG) and insulin levels were, respectively determined using the hexokinase method and the electrochemiluminescence immunoassay method. HOMA-IR and homeostatic model assessment of β-cell function (HOMA-β) indices were estimated from FPG and fasting insulin levels using the equations provided by Matthews et al. [21,22].

Inflammatory markers: Serum concentrations of high-sensitivity C-reactive protein (hs-CRP) and nitric oxide (NO) were determined using an immunoturbidimetric assay and enzyme linked immunosorbent assay, respectively. The levels of TNF-α, interleukin-6 (IL-6), interleukin-8 (IL-8), monocyte chemoattractant protein-1 (MCP-1), vascular endothelial growth factor (VEGF), leptin, vascular cell adhesion molecule-1 (VCAM-1), and intercellular adhesion molecule-1 (ICAM-1) were measured using a Human Luminex Discovery Assay Kit (R&D Systems, Minneapolis, MN, USA). All inter- and intra-assay coefficients of variation were below 5.0%.

#### 2.4.4. Dietary Records

For participants in CR group and CR–RS group, a self-designed APP was adopted to accurately record their food intake details. Diverse menus were included in the APP, and participants could keep a record of their food intake on it every day. Participants should clearly describe the ingredients, weight, volume, time, and place of each meal. Data were exported from the APP after the 8-week intervention.

For participants in RS group who did not receive any dietary intervention, three-day recalled dietary surveys were conducted at both allocation and the end of all intervention.

#### 2.4.5. Exercise Records

Smartwatches as well as smart-skipping ropes were provided for each participant during the study. Participants in RS group and CR–RS group kept a record of their exercise details by wearing smartwatches and using smart-skipping ropes.

For participants in CR group, three-day exercise records were required at both baseline and the end of intervention.

### 2.5. Quality Control

#### 2.5.1. Randomization

Stratified randomization was adopted at allocation. Both sex (male vs. female) and weight status (overweight vs. obesity) were taken into consideration during the randomization to make sure the groups were comparable.

#### 2.5.2. Participant Compliance

The self-designed APP was equipped with dietary and exercise alerts. Every day during the intervention, participants received alerts reminding them to report relevant data on the APP. In addition, Wechat groups were available for each group, where project workers could stay in touch with all participants.

### 2.6. Statistical Analyses

All data were input and double-checked by Epidata 3.1 and analyzed by SPSS software version 26 (IBM). Data normality was checked using the Shapiro–Wilk test. Continuous outcomes were log-transformed as needed to normalize distributions for analyses and presented as medians. Differences in baseline characteristics among the three groups were assessed with χ^2^ tests for categorical variables, and analysis of variance or Kruskal–Wallis tests for continuous variables. At the end of intervention, differences among groups were assessed with Analysis of Covariance, adjusting for baseline value (corrected for three comparisons using Bonferroni method); the lg-transformation for skewed variables was performed in those analyses, and means were then retransformed to the original scale to improve data interpretation. Within group differences were assessed using a paired sample *t*-test or Wilcoxon signed rank test. Additional analyses were performed in subgroup of overweight and obese participants. A *p*-value < 0.05 was considered as statistically significant.

## 3. Results

### 3.1. Study Overview and Participant Characteristics

A total of 46 participants were randomly assigned to the following three groups: CR group (*n* = 16), RS group (*n* = 15), and CR–RS group (*n* = 15). Two individuals in the CR group did not complete the intervention for poor compliance. One individual in the RS group withdrew due to an accidental knee injury. Three participants in the CR–RS group withdrew (one did not complete the intervention for poor compliance, and two were lost to follow-up). Overall, 40 participants completed the intervention and were included in the final analysis (14, 14, and 12 participants in the CR group, the RS group, and the CR–RS group, respectively) (Figure 1). There were no adverse events, including hunger, hypoglycemia, irritability, insomnia, or muscle soreness reported by participants during the 8-week trial.

The skipping rope intervention was under the supervision of a physical education (PE) teacher, and the median attendance rates of participants was 83%, which shows no significant difference between the RS group and the CR–RS group. Twenty-five out of the 26 participants who completed the CR intervention finished dietary records of three time points (baseline, 4 weeks, and 8 weeks). The dietary assessment data are presented in Table S1.

The baseline characteristics of the participants are shown in Table 1. The participants were aged 19.4 ± 0.6 years. The three groups were comparable at baseline with no significant difference in the distribution of age, sex, body mass index, body weight status, feeding patterns, mode of delivery, paternal and maternal educational level, monthly household income, and outdoor time.

### 3.2. Effect of Caloric Restriction and Exercise on Body Weight and Composition

Distribution of participants’ body weight and composition before and after the intervention are presented in Table 2. After 8 weeks of intervention, all three groups showed downward trends in body weight, BMI, body fat percentage (PBF), and body fat mass (BFM), although the RS group did not achieve statistical significance. The participants from the CR group and the CR–RS group reduced in body weight (−1.1 ± 1.7 kg, −1.3 ± 2.0 kg), BMI (−0.4 ± 0.6 kg/m^2^, −0.5 ± 0.7 kg/m^2^), PBF (−1.2 ± 1.6%, −1.7 ± 1.8%), and BFM (−1.1 kg (−2.2, −0.3), −1.1 kg (−2.5, −0.4)) (*p* < 0.05 or *p* = 0.051), while those from the RS group did not show a significant difference from baseline.

At the end of the intervention, no significant difference in body weight, BMI, PBF, or BFM was detected among the three groups.

### 3.3. Effect of Caloric Restriction and Exercise on Metabolic Risk Markers

The concentrations of the participants’ metabolic risk markers are presented in Table 3. After 8 weeks of intervention, the participants in the CR–RS group showed significant decreases in LDL-C (−0.40 mmol/L (−0.63, −0.16)), while no other significant difference from baseline was observed in the RS group or the CR group. At the end of the intervention, no significant difference in the assessed metabolic risk markers was detected among the three groups, including systolic blood pressure (SBP), diastolic blood pressure (DBP), TG, TC, HDL-C, LDL-C, FPG, insulin, HOMA-IR, β-cell function, and uric acid.

Subgroup analyses were performed for the participants with overweight or obesity at allocation (Table S2). The results showed that at the end of the intervention, the participants from the CR–RS group had significantly lower SBP and DBP levels than those in the other two groups (*p* < 0.05). In addition, the participants from the CR–RS group also showed levels of insulin and HOMA-IR lower than the participants from the CR group.

### 3.4. Effect of Caloric Restriction and Exercise on Inflammatory Markers

The concentrations of the participants’ inflammatory markers are presented in Table 4. After 8 weeks of intervention, all three groups showed downward trends in IL-6, IL-8, Leptin, and ICAM-1; however, only the participants in the CR–RS group had significant decreases in IL-8 (−0.73 pg/mL (−1.16, −0.29)). The participants from the CR group had a significant increase in MCP-1.

No significant difference from baseline was observed in hs-CRP, NO, TNF-α, IL-6, VEGF, Leptin, VCAM-1, and ICAM-1. At the end of the intervention, no significant difference in the assessed inflammatory markers was detected among the three groups.

For the participants with overweight or obesity at allocation (*n* = 23), sub-analysis among groups were performed (Table S3). The results showed that at the end of the intervention, the RS group had significantly lower hs-CRP levels than the CR group (*p* < 0.05), and both the RS group and the CR–RS group showed TNF-α levels lower than the CR group. In addition, the participants in the CR–RS group showed significantly lower IL-8 levels than those in the RS group.

## 4. Discussion

We aimed at evaluating the short-term effects of CR, RS, and their joint interventions on cardiometabolic health in young adults. We found that both the CR and CR–RS combined intervention could significantly reduce weight, BMI, PBF, and BFM in young adults, and the CR–RS combined intervention significantly reduced LDL-C and IL-8 levels, although no significant difference was found among the three groups. Importantly, in the subgroup analysis for the participants who already had overweight or obesity at baseline, the results showed the CR–RS combined intervention was superior to either CR or RS in improving SBP, DBP, IL-8, insulin, and HOMA-IR levels.

Although CR and exercise intervention were differently designed in various studies, prior results provided evidence that CR and exercise leads to weight loss and improvements in body composition [23]. In CALERIE phase one trials, Michael et al. demonstrated that 6 months of CR with or without exercise resulted in similar weight loss (10%) [10]. In the present study, we found that either 8 weeks of CR alone or a CR–RS combined intervention significantly reduced weight, BMI, PBF, and BFM in young adults, though the weight loss values were relatively limited, probably due to the short duration of the intervention (8 weeks). As indicated by a meta-analysis study, greater weight loss was found in studies lasting longer than 6 months, compared to studies designed with a shorter intervention time [24]. Future studies with sufficient time for intervention might lead to more significant results.

A few studies have investigated the effects of CR and exercise on cardiometabolic health [25,26,27]. Despite the inconsistent results and the vastly differently designed interventions that were in those studies, a few studies reported that an appropriate amount of exercise improved the cardiometabolic health in individuals with normal-weight, overweight, obesity, metabolic syndrome, or diabetes mellitus [28,29]. For instance, 12-week exercise training plus diet has been proven superior to diet alone in reducing triglycerides levels in overweight and obese individuals with type 2 diabetes mellitus [18]. In the present study, exercise intervention was designed as an RS exercise, and no effects of exercise intervention were found on the participants’ CV health. Although a few studies have demonstrated the improvement of LDL-C levels by CR or exercise intervention [23,30,31], in the present study, we only observed the improvement of LDL-C levels by the CR and RS combined intervention. The lack of improvement with CR or RS alone may be due to the short duration of the intervention in this study, but to some extent, it suggests that the combined intervention of CR and exercise may have a more significant effect on lipid levels [32]. Further studies are needed to explore the joint effect of CR and exercise on CV health.

Insulin resistance is an early risk factor for the development of several cardiometabolic diseases, including dyslipidemia, type 2 diabetes, metabolic syndrome, and coronary heart disease [33]. It is well established that CR and exercise improve insulin sensitivity in humans with overweight or obesity [25,34], while little significant effect of CR on insulin sensitivity in normal weight individuals was reported by previous studies. Similarly, our studies found that the improvement effects of the CR–RS combined intervention on fasting insulin and HOMA-IR levels were significantly superior to CR alone in overweight or obese individuals, but not in those with normal weight. In the present study, a few participants may have insulin resistance at baseline indicated by higher fasting insulin and HOMA-IR levels (despite no agreement on the cut-offs, fasting insulin > 8 mIU/L and HOMA-IR > 2 are suggested as two symptoms of insulin resistance by some researchers [35,36]), and most of them (~80%) were from the subgroup of those with overweight or obesity. This explains, in some ways, why the impact of diet and exercise interventions on insulin resistance was more obvious in the overweight and obese participants. Additionally, it is worth noting that some of the participants in this study had low FPG levels (lower than 3.9 mmol/L), which resulted in high values of HOMA-β according to the formula. The low FPG levels might partly be due to the situation of over fasting longer than 12 h, which needs to be further investigated.

Certain prior randomized controlled trials in adult humans have reported protective effects against inflammation, including reducing body fat mass, ameliorating oxidative stress, and modulating inflammatory cytokines levels in circulation [37,38,39]. In the present study, decreased IL-6, IL-8, Leptin, and ICAM-1 levels were found in all the participants after the 8-week intervention, indicating that strategies such as CR, RS, and CR–RS could, to some extent, improve the levels of inflammatory cytokines, though no significant difference was found among the three groups. On the other hand, in the sub-analysis for the participants who already had overweight or obesity at allocation, the CR–RS combined intervention showed significantly superior effects to CR or RS in ameliorating inflammation. The inconsistent results might indicate that the CR–RS combined intervention helped normalize inflammatory markers in young adults with excess weight, while its effects were limited and insignificant among adults within or under the normal weight range.

The strengths of the present study are that, to the best of our knowledge, this is the first to assess the influence of combining CR and RS exercise on inflammatory markers in young adults, and importantly, we found that the CR–RS combined intervention performed the best in reducing body weight, optimizing body composition, and improving CV health including LDL-C, IL-8, SBP, DBP, IL-8, insulin, and HOMA-IR levels in those with overweight or obesity. The present study, again, confirmed the protective effects of both CR and exercise on young adults’ CV health, and more importantly, proved that the combination of CR and exercise could provide better protective effects on CV health in young adults. For the limitations, this is a pilot study with a relatively small sample size and no blank control group, and we did not include the subgroup results of normal-weight participants, since the sub-group sample size was inadequate for detecting differences among groups. Hoping to minimize the effects of those confounders, a further study with an expanded sample size and a control group has been designed and is currently in progress.

## 5. Conclusions

The results of the present study showed that both CR and CR–RS intervention could decrease weight and BMI and improve body composition in young adults. However, for those who already have overweight and obesity, the CR–RS combined intervention may be superior to either CR or RS interventions in improving metabolic health and decrease the levels of proinflammatory markers, indicating that the CR–RS combined intervention might be a good way to improve the health status for young adults with excess weight.

## Figures and Tables

**Table 1:** The baseline characteristics of participants.

Variables	Total (*n* = 40)	CR (*n* = 14)	RS (*n* = 14)	CR–RS (*n* = 12)	*p* Value
Age, years, mean (SD)	19.4 (0.6)	19.4 (0.6)	19.3 (0.6)	19.4 (0.7)	0.840
Sex					0.938
Female	25 (62.5)	9 (64.3)	9 (64.3)	7 (58.3)	
Male	15 (37.5)	5 (35.7)	5 (35.7)	5 (41.7)	
Body weight status					0.794
Normal	17 (42.5)	7 (50.0)	5 (35.7)	5 (41.7)	
Overweight and Obese	23 (57.5)	7 (50.0)	9 (64.3)	7 (58.3)	
Feeding patterns					0.522
Breastfeeding	27 (73.0)	9 (64.3)	11 (84.6)	7 (70.0)	
Not breastfeeding	10 (27.0)	5 (35.7)	2 (15.4)	3 (30.0)	
Mode of delivery					0.133
Vaginal delivery	24 (64.9)	7 (50.0)	8 (61.5)	9 (90.0)	
Cesarean delivery	13 (35.1)	7 (50.0)	5 (38.5)	1 (10.0)	
Paternal educational level					0.836
Senior high school or below	15 (40.5)	5 (35.7)	5 (38.5)	5 (50.0)	
Junior college or above	22 (59.5)	9 (64.3)	8 (61.5)	5 (50.0)	
Maternal educational level					0.942
Senior high school or below	20 (54.1)	8 (57.1)	7 (53.8)	5 (50.0)	
Junior college or above	17 (45.9)	6 (42.9)	6 (46.2)	5 (50.0)	
Monthly household income					0.068
<5000 RMB/person	21 (56.8)	8 (57.1)	5 (38.5)	8 (80.0)	
≥5000 RMB/person	12 (32.4)	6 (42.9)	4 (30.8)	2 (20.0)	
Unknown	4 (10.8)	0 (0.0)	4 (30.8)	0 (0.0)	
Outdoor time					0.790
<1 h/d	16 (43.2)	6 (42.9)	6 (46.2)	4 (40.0)	
1~<2 h/d	17 (45.9)	6 (42.9)	5 (38.5)	6 (60.0)	
≥2 h/d	4 (10.8)	2 (14.3)	2 (15.4)	0 (0.0)	

Data are *n* (%). CR, calorie restriction; RS, rope-skipping; CR–RS, calorie restriction with rope-skipping. Differences among groups were assessed with χ^2^ tests or analysis of variance.

**Table 2:** Intervention effects on body weight and composition.

Variables	CR (*n* = 14)	RS (*n* = 14)	CR–RS (*n* = 12)	Among-Group *p*
Weight, kg				
Baseline ^a^	70.1 (11.5)	69.3 (11.5)	67.8 (9.6)	0.860
8 weeks ^a^	69.0 (11.5)	68.7 (11.6)	66.5 (8.4)	0.809
Change ^a^	−1.1 (1.7)	−0.6 (2.5)	−1.3 (2.0)	0.670
8-week-adjusted ^c^	68.1 (67.0, 69.1)	68.6 (67.5, 69.6)	67.8 (66.6, 68.9)	0.600
Within-group *p* ^d^	0.038	0.424	0.051	
BMI				
Baseline ^a^	24.8 (2.5)	24.4 (1.9)	24.5 (2.4)	0.914
8 weeks ^a^	24.4 (2.6)	24.2 (2.3)	24.1 (2.0)	0.937
Change ^a^	−0.4 (0.6)	−0.2 (1.0)	−0.5 (0.7)	0.721
8-week-adjusted ^c^	24.2 (23.8, 24.6)	24.3 (23.9, 24.7)	24.1 (23.7, 24.5)	0.710
Within-group *p* ^d^	0.046	0.434	0.049	
PBF, %				
Baseline ^a^	31.3 (7.0)	30.6 (7.7)	31.6 (7.9)	0.949
8 weeks ^a^	30.1 (7.5)	29.8 (7.9)	29.9 (8.3)	0.995
Change ^a^	−1.2 (1.6)	−0.8 (2.0)	−1.7 (1.8)	0.478
8-week-adjusted ^c^	29.9 (29.1, 30.8)	30.3 (29.4, 31.2)	29.4 (28.5, 30.4)	0.402
Within-group *p* ^d^	0.014	0.156	0.007	
BFM, kg				
Baseline ^b^	20.4 (17.8, 25.6)	20.5 (18.3, 23.0)	20.9 (17.2, 22.5)	0.969
8 weeks ^b^	19.8 (16.1, 24.7)	19.4 (15.3, 23.1)	19.6 (15.9, 22.0)	0.975
Change ^b^	−1.1 (−2.2, −0.3)	−0.3 (−1.3, 0.2)	−1.1 (−2.5, −0.4)	0.316
8-week-adjusted ^c^	19.5 (18.7, 20.4)	19.9 (19.1, 20.9)	19.2 (18.3, 20.1)	0.497
Within-group *p* ^d^	0.006	0.151	0.015	

CR, calorie restriction; RS, rope-skipping; CR–RS, calorie restriction with rope-skipping; BMI, Body mass index; PBF, Body fat percentage; BFM, Body fat mass. ^a^ Data are mean (SD), *p* values for among-group differences were calculated using analysis of variance. ^b^ Data are median (IQR), *p* values for among-group differences were calculated using Kruskal–Wallis test. ^c^ Data are mean (95% CI), *p* values for among-group differences were calculated using analysis of covariance, adjusting for baseline value. ^d^ *p* values for within-group differences were calculated using paired sample *t*-test or Wilcoxon signed rank test.

**Table 3:** Intervention effects on metabolic risk markers.

Variables	CR (*n* = 14)	RS (*n* = 14)	CR–RS (*n* = 12)	Among-Group *p*
SBP, mmHg				
Baseline ^a^	113 (10)	115 (8)	116 (12)	0.761
8 weeks ^a^	118 (11)	116 (11)	112 (12)	0.461
Change ^a^	5 (9)	1 (10)	−4 (7)	0.065
8-week-adjusted ^c^	119 (115, 123)	116 (111, 120)	111 (106, 116)	0.058
Within-group *p* ^d^	0.075	0.695	0.090	
DBP, mmHg				
Baseline ^a^	75 (9)	73 (5)	75 (8)	0.702
8 weeks ^a^	74 (7)	75 (7)	73 (10)	0.696
Change ^a^	0 (7)	2 (6)	−3 (7)	0.180
8-week-adjusted ^c^	74 (71, 77)	76 (73, 79)	72 (68, 75)	0.208
Within-group *p* ^d^	0.810	0.214	0.208	
TG, mmol/L				
Baseline ^b^	0.87 (0.63, 1.29)	0.84 (0.71, 1.05)	0.83 (0.67, 1.11)	0.993
8 weeks ^b^	0.94 (0.72, 1.21)	0.83 (0.72, 1.26)	0.97 (0.78, 1.24)	0.496
Change ^b^	0.08 (−0.08, 0.15)	−0.08 (−0.19, 0.17)	0.11 (−0.12, 0.60)	0.480
8-week-adjusted ^c^	0.97 (0.84, 1.12)	0.90 (0.78, 1.04)	1.11 (0.95, 1.30)	0.130
Within-group *p* ^d^	0.504	0.985	0.102	
TC, mmol/L				
Baseline ^a^	4.52 (0.70)	4.39 (0.73)	4.70 (0.85)	0.581
8 weeks ^a^	4.30 (0.58)	4.42 (0.89)	4.42 (0.54)	0.879
Change ^a^	−0.22 (0.58)	0.03 (0.65)	−0.29 (0.51)	0.358
8-week-adjusted ^c^	4.31 (4.05, 4.57)	4.50 (4.24, 4.76)	4.31 (4.02, 4.59)	0.492
Within-group *p* ^d^	0.188	0.872	0.080	
HDL-C, mmol/L				
Baseline ^a^	1.40 (0.41)	1.46 (0.31)	1.49 (0.29)	0.798
8 weeks ^a^	1.39 (0.30)	1.52 (0.28)	1.44 (0.28)	0.504
Change ^a^	−0.01 (0.20)	0.06 (0.15)	−0.06 (0.16)	0.239
8-week-adjusted ^c^	1.42 (1.35, 1.50)	1.51 (1.44, 1.59)	1.41 (1.33, 1.49)	0.114
Within-group *p* ^d^	0.796	0.158	0.268	
LDL-C, mmol/L				
Baseline ^a^	2.79 (0.61)	2.67 (0.64)	2.98 (0.79)	0.505
8 weeks ^a^	2.56 (0.46)	2.66 (0.83)	2.58 (0.62)	0.911
Change ^a^	−0.23 (0.51)	0.00 (0.63)	−0.40 (0.37)	0.172
8-week-adjusted ^c^	2.57 (2.33, 2.81)	2.75 (2.51, 2.99)	2.46 (2.20, 2.72)	0.267
Within-group *p* ^d^	0.119	0.980	0.004	
FPG, mmol/L				
Baseline ^a^	4.21 (0.58)	4.22 (0.30)	3.93 (0.38)	0.193
8 weeks ^a^	4.27 (0.50)	4.21 (0.36)	3.95 (0.42)	0.159
Change ^a^	0.06 (0.32)	0.00 (0.43)	0.02 (0.43)	0.915
8-week-adjusted ^c^	4.22 (4.04, 4.40)	4.16 (3.98, 4.34)	4.06 (3.87, 4.26)	0.520
Within-group *p* ^d^	0.496	0.981	0.881	
Insulin, mIU/L				
Baseline ^b^	10.59 (7.21, 14.42)	10.13 (8.00, 13.89)	7.93 (5.20, 11.44)	0.260
8 weeks ^b^	10.20 (7.73, 15.67)	11.36 (6.72, 14.04)	9.79 (6.94, 13.05)	0.630
Change ^b^	1.03 (−0.54, 2.75)	−1.04 (−3.77, 0.82)	−0.67 (−1.84, 2.24)	0.330
8-week-adjusted ^c^	13.07 (10.66, 15.48)	11.01 (8.60, 13.42)	10.18 (7.57, 12.78)	0.251
Within-group *p* ^d^	0.255	0.563	0.669	
HOMA-IR				
Baseline ^b^	1.89 (1.32, 2.91)	1.96 (1.48, 2.62)	1.38 (0.90, 2.02)	0.159
8 weeks ^b^	1.95 (1.26, 3.03)	2.06 (1.25, 2.80)	1.64 (1.13, 2.22)	0.491
Change ^b^	0.13 (−0.22, 0.43)	−0.13 (−0.64, 0.26)	−0.07 (−0.30, 0.39)	0.412
8-week-adjusted ^c^	2.53 (2.01, 3.05)	2.11 (1.59, 2.63)	1.82 (1.26, 2.39)	0.186
Within-group *p* ^d^	0.273	0.690	0.623	
HOMA-β				
Baseline ^a^	263.83 (188.64, 880.00)	280.19 (220.00, 370.40)	373.47 (266.07, 686.53)	0.566
8 weeks ^a^	367.63 (187.96, 748.70)	309.30 (236.87, 418.89)	370.47 (249.18, 758.90)	0.595
Change ^a^	52.03 (−73.93, 120.15)	−35.85 (−59.52, 62.71)	−41.38 (−169.59, 66.11)	0.690
8-week-adjusted ^c^	498.26 (293.20, 703.31)	552.72 (349.09, 756.34)	540.54 (324.13, 756.96)	0.931
Within-group *p* ^d^	0.889	0.959	0.842	
UA, μmol/L				
Baseline ^a^	390 (124)	390 (77)	406 (86)	0.904
8 weeks ^a^	377 (111)	358 (54)	375 (64)	0.785
Change ^a^	−13 (53)	−33 (66)	−30 (90)	0.727
8-week-adjusted ^c^	380 (351, 409)	360 (332, 389)	369 (338, 400)	0.639
Within-group *p* ^d^	0.376	0.086	0.268	

CR, calorie restriction; RS, rope-skipping; CR–RS, calorie restriction with rope-skipping; SBP, systolic blood pressure; DBP, diastolic blood pressure; TG, triglyceride; TC, total cholesterol; HDL-C, high density lipoprotein cholesterol; LDL-C, low density lipoprotein cholesterol; FPG, fasting plasma glucose; HOMA-IR, homeostatic model assessment of insulin resistance; HOMA-β, homeostatic model assessment of β-cell function; UA, uric acid. ^a^ Data are mean (SD), *p* values for among-group differences were calculated using analysis of variance. ^b^ Data are median (IQR), *p* values for among-group differences were calculated using Kruskal–Wallis test. ^c^ Data are mean (95% CI), *p* values for among-group differences were calculated using analysis of covariance, adjusting for baseline value. ^d^ *p* values for within-group differences were calculated using paired sample *t*-test or Wilcoxon signed rank test.

**Table 4:** Intervention effects on inflammatory markers.

Variables	CR (*n* = 14)	RS (*n* = 14)	CR–RS (*n* = 12)	Among-Group *p*
hs-CRP, mg/L				
Baseline ^b^	1.1 (0.6, 2.2)	0.5 (0.3, 1.1)	0.5 (0.2, 1.5)	0.203
8 weeks ^b^	1.2 (0.2, 1.7)	0.5 (0.3, 0.6)	0.35 (0.2, 1)	0.360
Change ^b^	−0.1 (−0.3, 0.2)	0.1 (−0.2, 0.2)	0.0 (−0.2, 0.3)	0.794
8-week-adjusted ^c^	0.6 (0.4, 1.0)	0.6 (0.3, 0.9)	0.6 (0.4, 1.1)	0.928
Within-group *p* ^d^	0.247	0.649	0.744	
NO, μmol/L				
Baseline ^b^	675.40 (142.75, 1000.00)	120.21 (74.44, 213.18)	34.27 (23.22, 256.87)	0.020
8 weeks ^b^	621.95 (516.87, 812.29)	97.68 (69.45, 123.29)	123.53 (37.29, 349.45)	0.028
Change ^b^	19.39 (0.00, 71.01)	0.15 (−8.18, 17.30)	−0.97 (−24.76, 19.14)	0.535
8-week-adjusted ^c^	178.67 (150.91, 211.54)	138.68 (114.49, 167.99)	141.71 (119.88, 167.52)	0.099
Within-group *p* ^d^	0.104	0.604	0.327	
TNF-α, pg/mL				
Baseline ^a^	2.05 (0.51)	2.04 (0.56)	2.23 (0.67)	0.664
8 weeks ^a^	2.22 (0.63)	1.95 (0.48)	2.05 (0.57)	0.457
Change ^a^	0.18 (0.68)	−0.09 (0.49)	−0.18 (0.48)	0.260
8-week-adjusted ^c^	2.25 (2.00, 2.49)	1.98 (1.72, 2.23)	1.99 (1.71, 2.26)	0.234
Within-group *p* ^d^	0.350	0.501	0.241	
IL-6, pg/mL				
Baseline ^a^	1.73 (0.95)	1.72 (1.07)	1.38 (0.32)	0.547
8 weeks ^a^	1.70 (0.60)	1.70 (1.02)	1.32 (0.24)	0.343
Change ^a^	−0.03 (0.92)	−0.02 (0.37)	−0.06 (0.26)	0.988
8-week-adjusted ^c^	1.64 (1.39, 1.89)	1.64 (1.38, 1.90)	1.46 (1.18, 1.75)	0.596
Within-group *p* ^d^	0.913	0.849	0.487	
IL-8, pg/mL				
Baseline ^a^	1.90 (0.54)	2.25 (0.91)	2.57 (1.42)	0.245
8 weeks ^a^	1.68 (0.47)	1.91 (0.51)	1.85 (1.37)	0.760
Change ^a^	−0.22 (0.69)	−0.34 (0.71)	−0.73 (0.65)	0.183
8-week-adjusted ^c^	1.87 (1.58, 2.17)	1.89 (1.59, 2.19)	1.62 (1.29, 1.95)	0.441
Within-group *p* ^d^	0.248	0.110	0.004	
MCP-1, pg/mL				
Baseline ^a^	54.50 (22.43)	63.92 (27.51)	67.91 (31.25)	0.442
8 weeks ^a^	74.34 (38.43)	80.17 (42.05)	74.01 (46.07)	0.917
Change ^a^	19.84 (27.32)	16.25 (37.06)	6.10 (26.15)	0.531
8-week-adjusted ^c^	81.90 (66.27, 97.53)	77.71 (61.69, 93.73)	67.30 (49.76, 84.84)	0.471
Within-group *p* ^d^	0.018	0.140	0.457	
VEGF, pg/mL				
Baseline ^a^	24.79 (11.15)	22.61 (11.35)	22.89 (10.42)	0.857
8 weeks ^a^	26.98 (13.84)	21.59 (11.14)	19.70 (10.87)	0.300
Change ^a^	2.19 (13.63)	−1.03 (13.79)	−3.19 (5.95)	0.534
8-week-adjusted ^c^	26.31 (20.89, 31.73)	22.04 (16.42, 27.65)	20.01 (13.91, 26.11)	0.292
Within-group *p* ^d^	0.558	0.793	0.106	
Leptin, ng/mL				
Baseline ^a^	12.44 (11.65)	13.05 (8.70)	12.46 (11.81)	0.987
8 weeks ^a^	12.41 (11.56)	11.94 (10.01)	8.54 (5.53)	0.571
Change ^a^	−0.02 (4.42)	−1.12 (5.86)	−3.93 (8.51)	0.307
8-week-adjusted ^c^	12.57 (9.77, 15.38)	11.65 (8.73, 14.56)	8.68 (5.51, 11.84)	0.179
Within-group *p* ^d^	0.984	0.505	0.157	
VCAM-1, ng/mL				
Baseline ^a^	383.15 (127.38)	452.66 (143.90)	451.60 (117.68)	0.304
8 weeks ^a^	397.65 (116.18)	450.39 (142.19)	498.31 (120.50)	0.156
Change ^a^	14.50 (87.75)	−2.27 (85.41)	46.71 (109.98)	0.446
8-week-adjusted ^c^	428.73 (384.51, 472.96)	431.91 (386.68, 477.15)	480.59 (431.49, 529.68)	0.240
Within-group *p* ^d^	0.547	0.925	0.189	
ICAM-1, ng/mL				
Baseline ^a^	142.29 (65.22)	129.79 (79.11)	125.95 (45.08)	0.804
8 weeks ^a^	134.44 (57.58)	123.94 (65.91)	122.70 (33.64)	0.836
Change ^a^	−7.85 (29.14)	−5.85 (18.86)	−3.25 (20.65)	0.890
8-week-adjusted ^c^	127.34 (117.67, 137.00)	126.69 (116.70, 136.68)	128.48 (117.60, 139.36)	0.972
Within-group *p* ^d^	0.332	0.285	0.613	

CR, calorie restriction; RS, rope-skipping; CR–RS, calorie restriction with rope-skipping; hs-CRP, high-sensitivity C-Reactive Protein; NO, nitric oxide; TNF-α, tumor necrosis factor-α; IL-6, interleukin-6; IL-8, interleukin-8; MCP-1, monocyte chemoattractant protein-1; VEGF, vascular endothelial growth factor; VCAM-1, vascular cell adhesion molecule-1; ICAM-1, intercellular adhesion molecule-1. ^a^ Data are mean (SD), *p* values for among-group differences were calculated using analysis of variance. ^b^ Data are median (IQR), *p* values for among-group differences were calculated using Kruskal–Wallis test. ^c^ Data are mean (95% CI), *p* values for among-group differences were calculated using analysis of covariance, adjusting for baseline value. ^d^ *p* values for within-group differences were calculated using paired sample *t*-test or Wilcoxon signed rank test.

## Data Availability

The data are not public.

## Supplementary Materials

The following are available online at https://www.mdpi.com/article/10.3390/nu13093222/s1, Table S1: Daily nutrients intake of participants, Table S2: Intervention effects on metabolic risk markers in participants with overweight or obesity, Table S3: Intervention effects on inflammatory markers in participants with overweight or obesity.
